# Post-marketing surveillance of tofacitinib in patients with ulcerative colitis in Japan: a final report of safety and effectiveness data

**DOI:** 10.1007/s00535-025-02249-5

**Published:** 2025-04-21

**Authors:** Katsuyoshi Matsuoka, Satoshi Motoya, Takayuki Yamamoto, Minoru Matsuura, Toshimitsu Fujii, Shinichiro Shinzaki, Yohei Mikami, Shoko Arai, Junichi Oshima, Yutaka Endo, Hirotoshi Yuasa, Masato Hoshi, Keiko Sato, Tadakazu Hisamatsu

**Affiliations:** 1 Division of Gastroenterology and Hepatology, Department of Internal Medicine, Toho University Sakura Medical Center, Chiba, Japan; 2 Clinical Epidemiology Committee of the Japanese Society for Inflammatory Bowel Disease, Shinjuku-ku, Tokyo, Japan; 3 Inflammatory Bowel Disease Center, Sapporo-Kosei General Hospital, Hokkaido, Japan; 4 Department of Surgery and IBD Center, Yokkaichi Hazu Medical Center, Yokkaichi, Mie Japan; 5 Department of Gastroenterology and Hepatology, Kyorin University, Mitaka, Tokyo Japan; 6 Department of Gastroenterology and Hepatology, Institute of Science Tokyo, Bunkyo-ku, Tokyo Japan; 7 Department of Gastroenterology, Faculty of Medicine, Hyogo Medical University, Nishinomiya, Hyogo Japan; 8 Division of Gastroenterology and Hepatology, Department of Internal Medicine, Keio University School of Medicine, Shinjuku-ku, Tokyo Japan; 9 Pfizer Japan Inc, Shibuya-ku, Tokyo Japan; 10 Pfizer R&D Japan, Shibuya-ku, Tokyo Japan

**Keywords:** Post-marketing surveillance, Tofacitinib, Ulcerative colitis

## Abstract

**Background:**

We present the final analysis of a tofacitinib post-marketing surveillance (PMS) study in Japanese patients with ulcerative colitis (UC).

**Methods:**

Safety/effectiveness data were evaluated (through Sept/30/2022). All patients with UC in Japan receiving tofacitinib were registered (60-week observation period). Adverse events (AEs) were recorded. Per protocol, several AEs were identified as clinically important/potential risks; all treatment-period data were used to calculate incidence rates (IRs; unique patients with events/100 patient-years [PY] of exposure). Effectiveness was assessed (partial/total Mayo score), with last observation carried forward for imputation of missing data.

**Results:**

Overall, 2043 patients were enrolled (safety analysis set: *n* = 1982/effectiveness analysis set: *n* = 1969). Data were excluded for 13 patients from two hospitals from which consent was not obtained for publication and which, therefore, were not permitted for publication. AEs and serious AEs were observed in 33.4% and 5.2% of patients, respectively; one death occurred (intestinal abscess). Herpes zoster (HZ; non-serious and serious) was the most reported infection (*n* = 92 [IR 5.93/100 PY, 95% confidence interval 4.78, 7.27]). Serious infection, malignancy, cardiovascular and venous thromboembolic events IRs were 1.51/100 PY, 0.62/100 PY, 0.13/100 PY, and 0.31/100 PY, respectively. Overall, 52.4% of patients discontinued treatment, mostly due to inadequate clinical response (48.9%). At Week 60, 1151/1969 patients (58.5%) achieved partial Mayo score remission.

**Conclusion:**

The overall safety profile was generally comparable with tofacitinib data from PMS reports from Japan, worldwide and the tofacitinib UC clinical program. However, HZ IR was higher than in the tofacitinib UC clinical program. Tofacitinib effectiveness was consistent with data from the tofacitinib UC clinical program.

**ClinicalTrials.gov:**

NCT03643211.

**Supplementary Information:**

The online version contains supplementary material available at 10.1007/s00535-025-02249-5.

## Introduction

Ulcerative colitis (UC) is a chronic inflammatory bowel disease, primarily affecting the colon and rectum; UC has a heterogenous symptom presentation that includes rectal bleeding and abdominal pain [1, 2]. While western countries were once considered to have the highest global prevalence of UC (e.g., highest estimated incidence in Northern Europe [57.9 per 100,000 people] and North America [23.1 per 100,000 people]) [3], the incidence of UC is now also increasing in Asia [4]. A recent systematic analysis of the global prevalence of inflammatory bowel disease observed that the highest age-standardized prevalence rate was observed in the high-income Asia–Pacific region, which includes Japan (210.54 per 100,000 people) [2].

Tofacitinib is an oral Janus kinase inhibitor for the treatment of UC. Among Japanese or East-Asian patients with moderate to severe UC, safety and effectiveness of tofacitinib have been evaluated from two identical 8-week Phase 3 induction studies (OCTAVE Induction 1 and 2) [5–7], a 52-week Phase 3 maintenance study (OCTAVE Sustain) [5–7] and an open-label, long-term (up to 7.0 years) extension study (OCTAVE Open) [8, 9].

As only a low number of patients from Japan were included in previous clinical studies, and because post-marketing information on the drug is collected for a pre-determined period after approval in Japan, a post-marketing surveillance (PMS) study was conducted following approval of tofacitinib in Japan, for all patients who received tofacitinib for the treatment of UC.

Here, we present the final analysis of the PMS study of tofacitinib when administered long term to patients with UC in a real-world setting. This PMS study examined the safety and effectiveness of tofacitinib in a post-marketing clinical setting, based on the conditions for approval.

## Materials and methods

### Patients and study design

A final analysis of safety and effectiveness data from a PMS study was conducted (data as of September 30, 2022). The study was conducted between May 25, 2018 and June 30, 2021, and was conducted as all-case surveillance. All Japanese patients with UC receiving tofacitinib were prospectively registered in the study.

The observation period was defined as the tofacitinib treatment period, which was the period from the date of tofacitinib initiation up to 60 weeks after the start date or up to June 2021. Patients who were treated with tofacitinib for more than 60 weeks after the date of treatment commencement were continuously observed until June 2021 (end of observation period) so that incidence of malignancies could be recorded for the longest period possible. For patients who discontinued tofacitinib treatment, data were collected from treatment commencement up to the point of discontinuation.

Data were collected from sites as electronic or paper-based case report forms, which were later updated through queries (sometimes regarded as data clarification forms) to highlight and address discrepancies and errors.

### Analysis of safety

Tofacitinib safety was evaluated in patients included in the safety analysis set, defined as patients confirmed to have received at least one dose of tofacitinib. Assessments of safety included adverse events (AEs; defined as any unfavorable event occurring after the administration of tofacitinib, whether related or not) or adverse drug reactions (ADRs; defined as AEs for which a causal relationship to the drug could not be ruled out) that occurred during the observation period (tofacitinib treatment period, from treatment initiation up to 60 weeks after the start date or the end date of the observation period, whichever came sooner). AEs were classified as serious if they resulted in death, were life threatening, required inpatient hospitalization or prolongation of existing hospitalization, resulted in persistent or significant disability/incapacity, were a congenital anomaly/birth defect, or any other medically important event that may have led to disability. All AEs and ADRs were recorded during tofacitinib treatment. For malignancy, ‘AEs’ referred to those that occurred until the end of the observation period, regardless of start date of administration (i.e., more than 60 weeks).

The causal relationship and seriousness of AEs were tabulated based on the investigator’s assessment. AEs were coded to the Preferred Term and System Organ Class using the Medical Dictionary for Regulatory Activities (MedDRA) version 24.1. For calculation of the number of episodes of AEs, multiple occurrences of the same Preferred Term event in the same patient were considered as ‘1 event’.

In the risk-management plan, several AEs were identified as clinically important risks or as potential risks. These were: ‘serious infections’ (including all infections classified as serious and coded as MedDRA System Organ Class ‘Infections and Infestations’, except herpes zoster [HZ]), ‘HZ’, ‘neutropenia, lymphocytes decreased, hemoglobin decreased’, ‘hepatic function disorder’, ‘reactivation of hepatitis B virus’, ‘gastrointestinal perforation’, ‘interstitial lung disease’, ‘venous thromboembolism’, ‘malignancy’, ‘cardiovascular events’ and ‘rhabdomyolysis and myopathy’.

### Effectiveness assessments

The effectiveness analysis set consisted of patients from the safety analysis set for whom effectiveness was reported. Tofacitinib effectiveness was evaluated using the partial Mayo score (primary assessment of effectiveness [0–9 points]), which is a measure of disease activity comprising three subscores (0–3 points): stool frequency, rectal bleeding, and Physician Global Assessment (PGA) [10]. Effectiveness was also measured using the total Mayo score (secondary assessment of effectiveness [0–12 points]) which includes the same disease activity measures as the partial Mayo score, with the addition of an endoscopic assessment of the severity of colonic inflammation (Mayo endoscopic subscore). Higher partial Mayo/total Mayo scores indicate more severe disease. Remission based on the partial Mayo score was defined as a partial Mayo score of ≤ 2, stool frequency subscore of ≤ 1, rectal bleeding subscore of ≤ 1, and PGA subscore of ≤ 1. The partial Mayo score was measured during the observation period at baseline and weeks 2, 4, 8, 12, 16, 24, 32, 40, 48, and 60. Remission by total Mayo score was defined as a total Mayo score of ≤ 2, stool frequency, PGA and endoscopic subscores of ≤ 1, and rectal bleeding subscore of 0. The total Mayo score was measured at weeks 8, 16, 24, 32, 40, 48, and 60***.*** All data were evaluated as observed (without imputation of missing data) and with last observation carried forward (LOCF) for imputation of missing data, specifically non-responder imputation (NRI) if there were no data to impute (hereafter NRI-LOCF).

### Statistical analysis

The main analysis items were the occurrence of the AEs of interest (clinically important/potential risks), incidence of AEs of interest (number of patients with AEs/total number of patients in the safety analysis set, expressed as a percentage), and incidence of AEs of interest per duration of exposure (100 × number of patients with AEs/total duration of exposure, expressed as an incidence rate [IR] per 100 patient-years [PY]). In addition, baseline demographics affecting the occurrence of AEs of interest were also examined by analyzing subpopulations by patient characteristics.

In the risk-management plan, for the AEs that were identified as clinically important risks or as potential risks, all treatment-period data were used to calculate cumulative IRs (unique patients with events per 100 PY of exposure). Proportions and IRs (95% confidence intervals [CIs]) were also evaluated for ADRs.

### Ethical considerations

Ethics committee approval was exempt as this was a PMS. This study was an all-case surveillance, non-interventional study based on the local regulations in Japan. Patient consent was not required.

## Results

### Baseline demographics and clinical characteristics

In total, 2043 patients from 456 sites received tofacitinib and were enrolled in the PMS study, of whom 2003 had their case report form fixed. Data were excluded for 13 patients from two hospitals from which consent was not obtained and which were, therefore, not permitted for publication. Of those patients, 1982 and 1969 were included in the safety and effectiveness analysis sets, respectively (Supplementary Fig. 1). Mean age was 42.5 years and mean partial and total Mayo scores were 5.4 and 7.8, respectively. In total, 6.2% of patients were current smokers and 62.7% had never smoked (Table 1). Most patients had prior biologic use (69.0%) and approximately half had been treated with corticosteroids before starting tofacitinib (49.4%).

**Table 1:** Baseline demographics and clinical characteristics of patients in the PMS study

	Safety analysis set (*N* = 1982; 2706.4 PY)
Age (years), mean (SD)	42.5 (16.2)
Age, *n* (%)	
< 15 years^a^	12 (0.6)
≥ 15 to < 65 years	1758 (88.7)
≥ 65 years	212 (10.7)
Sex, *n* (%)	
Male	1199 (60.5)
Female	778 (39.3)
Unknown	5 (0.3)
Partial Mayo score	
Mean (SD)	5.4 (1.9)^b^
Median	6.0
Total Mayo score	
Mean (SD)	7.8 (2.1)^c^
Median	8.0
Disease duration (years), mean (SD)	7.9 (7.5)
Extent of disease, *n* (%)	
Extensive colitis	1456 (73.6)
Left-sided colitis	458 (23.2)
Proctitis	49 (2.5)
Right-sided or segmental colitis	6 (0.3)
Other	9 (0.5)
Prior biologic use, *n* (%)^d^	1367 (69.0)
Infliximab	820 (60.0)^e^
Adalimumab	583 (42.6)^e^
Golimumab	557 (40.7)^e^
Other^f^	182 (13.3)^e^
Prior corticosteroid use, *n* (%)^g^	979 (49.4)^e^
Prior 5-aminosalicylates use, *n* (%)^g^	1587 (80.1)
Prior immunomodulator use, *n* (%)^g,h^	587 (29.6)
Smoking status, *n* (%)	
Current smoker	122 (6.2)
Never smoked	1242 (62.7)
Former smoker	380 (19.2)
Unknown	238 (12.0)

In total, 1982 patients were included in the safety analysis set

^a^Tofacitinib is not approved for use in children in Japan

^b^*N1* = 1598

^c^*N1* = 262

^d^Patients may have been receiving combination biologic therapy

^e^Percentage calculated using the total number of patients with prior biologic use at baseline as the denominator

^f^Ustekinumab, vedolizumab, etanercept, tocilizumab. Ustekinumab and vedolizumab were not approved in Japan when the PMS study was initiated; therefore, more detailed analysis of prior treatment with these drugs was not possible

^g^History of treatment for the episode immediately before the commencement of tofacitinib treatment

^h^Azathioprine or 6-mercaptopurine

*n* number of patients with characteristic, *N* number of patients evaluated, *N1* number of patients evaluated for the characteristic, *PMS* post-marketing surveillance, *PY* patient-years, *SD* standard deviation

### Analysis of safety

AEs and serious AEs were observed in 661 (33.4%) and 103 (5.2%) patients, respectively.

HZ (non-serious and serious) was the most reported AE of interest (92 patients [4.6%], IR 5.93 [95% CI 4.78, 7.27]; Table 2). In total, serious HZ was reported in four patients (0.2%) and there were no cases of disseminated HZ. Dyslipidemia was reported in 90 patients (4.5% [IR 5.90; 95% CI 4.74, 7.25]), decreased neutropenia/lymphopenia/hemoglobin was reported in 54 patients (2.7% [IR 3.46; 95% CI 2.60, 4.51]), and serious infections were reported in 24 patients (1.2% [IR 1.51; 95% CI 0.97, 2.25]). Malignancies were reported in 17 patients (0.9% [IR 0.62; 95% CI 0.36, 1.00]), which included three cases of colon cancer and two cases of neoplasm malignant and testis cancer, among others. Venous thromboembolism was reported in five patients (0.3% [IR 0.31; 95% CI 0.10, 0.73]) and cardiovascular events were reported in two patients (0.1% [IR 0.13; 95% CI 0.02, 0.45]). The proportion and IRs of ADRs in the PMS study are reported in Supplementary Table 1. There was no significant difference in the IR of HZ when stratified by time to onset (Table 3).

**Table 2:** Proportions and IRs of AEs of interest in the PMS study^a^

Clinically important/potential risks^b^	AE^b^		
	*n* (%)	Exposure, PY	IR (95% CI)
HZ (non-serious and serious)	92 (4.6)^c^	1551.56	5.93 (4.78, 7.27)
Dyslipidemia	90 (4.5)^d^	1526.34	5.90 (4.74, 7.25)
Neutropenia/lymphopenia/hemoglobin decreased	54 (2.7)^e^	1561.07	3.46 (2.60, 4.51)
Rhabdomyolysis/myopathy	24 (1.2)^f^	1576.88	1.52 (0.98, 2.26)
Serious infection	24 (1.2)^g^	1587.49	1.51 (0.97, 2.25)
Liver dysfunction	28 (1.4)^h^	1575.84	1.78 (1.18, 2.57)
Malignancy	17 (0.9)^i^	2729.70	0.62 (0.36, 1.00)
Venous thromboembolism	5 (0.3)^j^	1592.23	0.31 (0.10, 0.73)
Interstitial lung disease	6 (0.3)^k^	1591.51	0.38 (0.14, 0.82)
Gastrointestinal perforation	6 (0.3)^l^	1592.32	0.38 (0.14, 0.82)
Reactivation of hepatitis B virus	0^ m^	19.75	0.00^n^
Cardiovascular events^o^	2 (0.1)^p^	1593.79	0.13 (0.02, 0.45)

All events were non-adjudicated and judged by the investigators. The observation period was defined as the tofacitinib treatment period, which was the period from the date of tofacitinib initiation up to 60 weeks after the start date or up to June 2021. Patients who were treated with tofacitinib for more than 60 weeks after the date of treatment commencement were continuously observed until June 2021 (end of the observation period). All AEs within 60 weeks or until the end of the observation period (whichever came sooner) were recorded. Malignancies were recorded until the end of the observation period

^a^Data reported during the treatment period; 1982 patients were included in the safety analysis (investigator-reported Preferred Term MedDRA v.24.1)

^b^An AE was defined as any unfavorable event (including a clinically significant abnormal laboratory change) occurring after administration of tofacitinib, whether related to tofacitinib or not

^c^Four events (0.2%) were serious

^d^Metabolism and nutrition disorders: hyperlipidemia (*n* = 30); dyslipidemia (*n* = 20); hypercholesterolemia (*n* = 20); and hypertriglyceridemia (*n* = 6). Congenital, familial and genetic disorders: type IIa hyperlipidemia (*n* = 1). Investigations: lipids abnormal (*n* = 5); blood cholesterol increased (*n* = 4), low-density lipoprotein increased and blood triglycerides increased (both *n* = 3)

^e^Blood and lymphatic system disorders: anemia (*n* = 28); and anemia macrocytic (*n* = 1). Investigations: lymphocyte count decreased (*n* = 12); neutrophil count decreased (*n* = 9); hemoglobin decreased (*n* = 5) and red blood count cell decreased (*n* = 1)

^f^Musculoskeletal and connective tissue disorders: rhabdomyolysis (*n* = 1). Investigations: blood creatine phosphokinase increased (*n* = 23)

^g^Infections and infestations: HZ (*n* = 4); cytomegalovirus infection, cytomegalovirus enterocolitis, pyelonephritis, pneumonia and atypical mycobacterial infection (all *n* = 2); and clostridium difficile colitis, clostridium colitis, cytomegalovirus chorioretinitis, eczema herpeticum, infection, enteritis infectious, diverticulitis, bone tuberculosis, appendicitis, abscess intestinal and sepsis (all *n* = 1)

^h^Hepatobiliary disorders: liver disorder (*n* = 15) and hepatic steatosis (*n* = 3). Investigations: alanine aminotransferase increased and aspartate aminotransferase increased (both *n* = 5); and hepatic enzyme increased (*n* = 2)

^i^Colon cancer (*n* = 3); neoplasm malignant and testis cancer (both *n* = 2); and Epstein–Barr virus-associated lymphoproliferative disorder, lymphoma, hepatocellular carcinoma, basal cell carcinoma, cervix carcinoma, esophageal carcinoma, adenocarcinoma, pancreatic carcinoma and pancreatic carcinoma state II (all *n* = 1). Investigations: carcinoembryonic antigen increased and Krebs von den Lungen-6 increased (both *n* = 1)

^j^Venous thromboembolism events were identified using Preferred Terms in the Standardized MedDRA ‘Embolic and thrombotic events, venous’: thrombophlebitis and venous thrombosis limb (both *n* = 2) and deep vein thrombosis (*n* = 1)

^k^Respiratory, thoracic and mediastinal disorders: interstitial lung disease (*n* = 4); and eosinophilic pneumonia and hypersensitivity pneumonitis (both *n* = 1)

^l^Gastrointestinal disorders: anal fistula (*n* = 2); and rectal perforation (*n* = 1); Infections and infestations: appendicitis, abscess intestinal, diverticulitis and anal abscess (all *n* = 1)

^m^Only patients with a history of hepatitis B virus or complications of hepatitis B virus carrier were included (*n* = 23); ‘Exposure, PY’ is based on these patients only

^n^95% CI not calculated

^o^Cardiovascular events (excluding hyperlipidemia and lipid increase) were defined as ≥ 1 of the following: Standardized MedDRA ‘cardiac death’, ‘central nervous system vascular disease (narrow zone)’, ‘congestive heart failure’, ‘myocardial infarction (narrow zone)’, ‘other ischemic heart disease (narrow zone)’, ‘pulmonary embolism’, and ‘sudden cardiac death

^p^Nervous system disorders: subarachnoid hemorrhage (*n* = 1); injury, poisoning and procedural complications: subdural hematoma (*n* = 1)

*AE* adverse event, *CI* confidence interval, *HZ* herpes zoster, *IR* incidence rate (number of unique patients with events per 100 PY of exposure), *MedDRA* Medical Dictionary for Regulatory Activities, *n* number of patients with the AEs of interest, *n* number of cases, *PMS* post-marketing surveillance, *PY* patient-years

**Table 3:** Time to onset of HZ AE (weeks) in the PMS study^a^

	IR (95% CI)
Time to onset of HZ AE (weeks)	
Overall	5.93 (4.78, 7.27)
≤ 15	7.27 (5.06, 10.11)
> 15 to ≤ 30	4.77 (2.87, 7.45)
> 30 to ≤ 45	5.99 (3.71, 9.16)
> 45 to ≤ 60	5.29 (3.08, 8.47)

^a^Data reported during the treatment period, as of September 30, 2022; 1982 patients were included in the safety analysis set

*AE* adverse event, *CI* confidence internal, *HZ* herpes zoster, *IR* incidence rate (number of unique patients with events per 100 PY of exposure), *PMS* post-marketing surveillance, *PY* patient-years

Risk factor analysis showed that certain baseline demographics and disease characteristics may have increased the risk of a HZ AE (Table 4). Having a previous infection was the factor most likely to increase the risk of a HZ AE (risk ratio 4.72 [95% CI 2.72, 8.19]). Older age (≥ 65 vs < 50 years; risk ratio 2.06 [95% CI 1.21, 3.49]) was also associated with an increased risk of a HZ AE. Risk factor analysis results for association of each baseline variable with HZ AEs (ADRs) in the PMS study can be found in Supplementary Table 2.

**Table 4:** Risk factor analysis results for association of each baseline variable with HZ AEs in the PMS study^a^

Factor	Risk ratio (95% CI)
Sex	
Female vs male	1.13 (0.76, 1.70)
Age	
≥ 50 to < 65 years vs < 50 years	1.34 (0.83, 2.15)
**≥ 65 years vs < 50 years**	**2.06 (1.21, 3.49)**
Duration of ulcerative colitis	
≥ 2 to < 5 years vs < 2 years	1.01 (0.52, 1.93)
≥ 5 to < 10 years vs < 2 years	1.31 (0.70, 2.44)
≥ 10 to < 20 years vs < 2 years	1.74 (0.95, 3.16)
≥ 20 years vs < 2 years	0.98 (0.39, 2.45)
Smoking history	
Former smoker vs never smoked	1.43 (0.90, 2.27)
Current smoker vs never smoked	1.11 (0.49, 2.53)
History of infection^b^	
**Yes vs no**	**4.72 (2.72, 8.19)**
Prior corticosteroid use^c^	
Yes vs no	1.02 (0.69, 1.53)
Prior biologic use	
Yes vs no	1.62 (1.00, 2.63)

AEs that occurred between the date of commencement of treatment and week 60 in this study or the date of completion of the observation period, whichever came first, were included. Risk ratio was calculated as the ratio between the incidence of HZ in each of the categories being compared, e.g., incidence of HZ in females/incidence of HZ in males. 95% CIs were calculated using asymptotic method. Factors associated with an increased risk of HZ in the risk factor analysis are shown in bold text

*AE* adverse event, *CI* confidence interval, *HZ* herpes zoster, *PMS* post-marketing surveillance

^a^Data reported during the treatment period; 1982 patients were included in the safety analysis set

^b^Included HZ and all other infections

^c^History of treatment for the episode immediately before the commencement of tofacitinib treatment

### Discontinuations in the PMS study

**Fig. 1:**
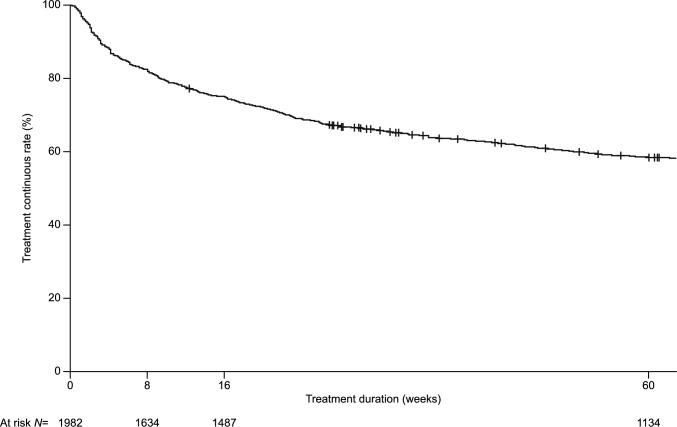
Summary of treatment continuation in the PMS study^a^. ^a^Data reported during the treatment period. *PMS* post-marketing surveillance

Overall, 1038/1982 patients (52.4%) discontinued tofacitinib treatment; approximately 75% of patients continued treatment to week 16, with approximately 50% continuing to week 60 (Fig. 1). The most common reason for treatment discontinuation was inadequate clinical response (508/1038 patients [48.9%]; Table 5). The second most common reason was AEs (193/1038 patients [18.6%]). Throughout the PMS study, one patient discontinued due to an unknown reason and one patient died following an intestinal abscess (not related to study drug, per investigator’s assessment).

**Table 5:** Reasons for discontinuations in the PMS study^a^

Reasons for discontinuation, *n* (%)^b^	Duration of treatment					
	≤ 8 weeks	> 8 to ≤ 16 weeks	> 16 to ≤ 26 weeks	> 26 to ≤ 60 weeks	> 60 weeks	Overall
AEs	88 (24.6)	24 (16.9)	30 (21.6)	24 (11.7)	27 (13.9)	193 (18.6)
Death	0	0	1 (0.7)	0	0	1 (0.1)
Remission	4 (1.1)	7 (4.9)	11 (7.9)	21 (10.2)	20 (10.3)	63 (6.1)
Inadequate clinical response	216 (60.5)	79 (55.6)	62 (44.6)	95 (46.1)	56 (28.9)	508 (48.9)
Lost to follow-up	14 (3.9)	14 (9.9)	13 (9.4)	32 (15.5)	43 (22.2)	116 (11.2)
Other	35 (9.8)	17 (12.0)	22 (15.8)	34 (16.5)	48 (24.7)	156 (15.0)
Unknown	0	1 (0.7)	0	0	0	1 (0.1)

^a^Data reported during the treatment period; 1982 patients were included in the safety analysis set

^b^Includes duplicates. Percentages were calculated based on the number of patients who discontinued treatment in each period

*AE* adverse event, *n* number of patients who discontinued, *PMS* post-marketing surveillance

### Analysis of effectiveness

**Fig. 2:**
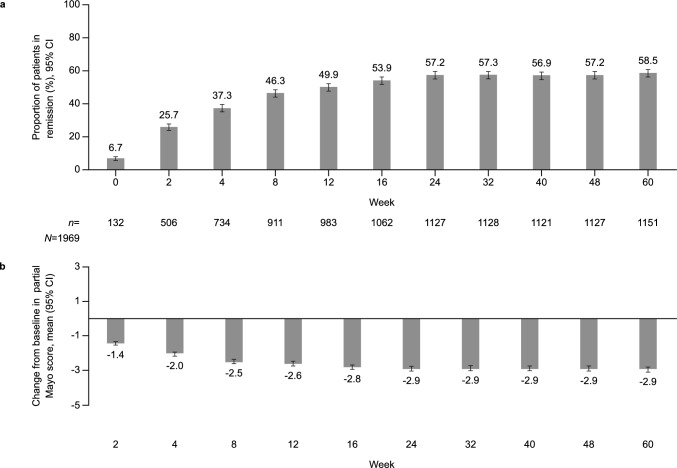
**a** Proportion of patients in remission by partial Mayo score and **b** mean changes from baseline in partial Mayo score, up to week 60 in the PMS study^a^ (NRI-LOCF). The observation period was defined as the tofacitinib treatment period, which was the period from the date of tofacitinib initiation up to 60 weeks after the start date or up to June 2021. Patients who were treated with tofacitinib for more than 60 weeks after the date of treatment commencement were continuously observed until June 2021 (end of the observation period). Remission by partial Mayo score was defined as Mayo score of ≤ 2 points, stool frequency subscore of ≤ 1 point, rectal bleeding subscore of ≤ 1 point, and PGA subscore of ≤ 1 point. The remission rate was defined as the proportion of patients in remission among those with evaluable partial Mayo score in the effectiveness analysis set (NRI-LOCF). Calculation of change from partial baseline Mayo score was based on the number of patients for whom the amount of change in partial Mayo scores could be calculated. Patients in whom a value at the treatment commencement was available, and Mayo score value for at least one time point after the treatment commencement was available were included (*n* = 1596 and *n* = 1191, respectively). ^a^Data reported during the treatment period. *CI* confidence interval, *n* number of patients in remission by partial Mayo score, *N* number of patients included in the effectiveness analysis set, *NRI-LOCF* non-responder imputation-last observation carried forward, *PGA* Physician Global Assessment, *PMS* post-marketing surveillance

The proportion of patients in remission by partial Mayo score as well as mean changes from baseline in the PMS study are shown in Fig. 2 (NRI-LOCF) and by partial Mayo score and total Mayo score in Supplementary Fig. 2 (as observed). Of note, NRI-LOCF values for total Mayo score were not reported due to the low number of patients with recorded endoscopic subscores. At week 60, 1151 of 1969 patients (58.5%) were in partial Mayo score remission (Fig. 2A). The mean change in partial Mayo score was – 1.4 at week 2 and – 2.9 at week 60 (Fig. 2B).

## Discussion

Overall, the final results of this PMS study in tofacitinib-treated patients with UC from Japan in a real-world setting were consistent with the previously published global studies in patients with UC treated with tofacitinib, except for generally lower rates of serious AEs and discontinuations [8, 11], and were also consistent with data from East Asian tofacitinib-treated patients with UC in the OCTAVE Open study [9].

In this study, AEs were observed in 661/1982 (33.4%) of patients. Previous studies investigating the safety of other Janus kinase inhibitors, and biologics in Japanese patients with UC have shown numerically higher rates of AEs when compared with tofacitinib; specifically, treatment emergent AEs for filgotinib 100 mg, filgotinib 200 mg, adalimumab 40 mg every week, and adalimumab 40 mg every other week were reported in 23/45 (51.1%), 24/44 (54.5%), 41/46 (89.1%), and 37/43 (86.0%) of patients, respectively [12, 13].

Of the AEs of interest assessed in this PMS study, serious infections were reported in a relatively small number of patients; HZ was the most reported infection and most cases were non-serious. These findings are consistent with previous findings that HZ (serious and non-serious) was the most commonly reported AE of interest in multiple global studies [8, 14, 15], and that this HZ risk appears higher in Asian versus non-Asian patients with autoinflammatory conditions [11, 15, 16]. Of note, the HZ IR reported in this study (IR 5.93 [95% CI 4.78, 7.27; 1551.56 PY of exposure]) was higher than that reported in patients with UC who received corticosteroids, immunosuppressants, or tumor necrosis factor inhibitors in the Medical Data Vision and Japan Medical Data Center databases (IR 1.00 [95% CI 0.85, 1.16; 16285 PY of exposure] and IR 1.82 [95% CI 1.27, 2.37; 2310.67 PY of exposure]), respectively [17]. However, the HZ IR reported here was lower than that reported in patients with rheumatoid arthritis in a PMS study in Japan (IR 8.02 [95% CI 7.05, 9.09; 3054.4 PY of exposure]) [18]. Moreover, the serious infection IR reported in this study (IR 1.51 [95% CI 0.97, 2.25; 1587.49 PY of exposure]) was numerically lower than the rates of hospitalized infections in the Medical Data Vision database (IR 1.73 [95% CI 1.52, 1.93; 16115 PY of exposure]) [17] and in the PMS study of patients with rheumatoid arthritis (IR 6.91 [95% CI 6.01, 7.91; 3066.2 PY of exposure]) [18]. These data are a reminder to healthcare professionals to continue to weigh risks against benefits when using tofacitinib or other advanced therapies.

The duration from tofacitinib treatment to the onset of HZ AE was considered to have no significant effect on the IR. However, risk factor analysis suggested that there were some baseline demographic and disease characteristics that may increase the risk of a HZ AE. These included previous infections and age ≥ 65 years versus < 50 years. The most common previous infection was herpes zoster (*n* = 28, 1.4%), and other infections included hepatitis B, and pneumonia. A recent retrospective cohort study in patients with UC and Crohn’s disease demonstrated that the HZ IR was higher in patients with versus without prior biologic exposure [19]. A post hoc analysis of data from this PMS study which focuses on patients with a history of HZ, tofacitinib dose at event onset, patients who started on low dose of tofacitinib (10 mg/day) and the difference in HZ incidence among sex, smoking status, and body mass index may provide further insight into the HZ risk in Japanese patients with UC. In addition, in a study of patients with UC from the tofacitinib UC clinical program, it was found that patients who were older, had prior tumor necrosis factor inhibitor failure, and were Asian had the highest HZ risk [16].

One of the reasons that patients with UC in Japan and Asia are at an increased risk of HZ may be their genetic background. A previous genome-wide association study meta-analysis in patients with rheumatoid arthritis or psoriasis treated with tofacitinib found that genes which confer susceptibility to HZ are different between European and East Asian patients. For example, the frequency of a risk allele was found to be higher in East Asian versus European patients (~ 12% vs < 0.2%) [20]. To help reduce the risk of patients with UC developing HZ, multiple vaccines are available, including the varicella vaccine live vaccine and the adjuvanted recombinant zoster vaccine [21]. Both vaccines are approved for use in Japan; however, live vaccines such as the varicella vaccine live are typically contraindicated for patients undergoing immunosuppressive therapy. While the recombinant zoster vaccine can be administered to these patients, it is still largely unknown if the vaccine reduces the risk of HZ in patients taking Janus kinase inhibitors [22].

The types of malignancies reported in this PMS study were similar to those reported in PMS studies of patients with other autoinflammatory conditions. Here, a total of 17 patients reported malignancies (IR 0.62 [95% CI 0.36, 1.00]; 2729.70 PY of exposure), with the most reported being colon cancer (three cases), neoplasm malignant (two cases), and testis cancer (two cases). There were also reports of lymphoma, lymphoproliferative disorder, carcinoma (hepatocellular, basal cell, cervix, esophageal, and pancreatic), and adenocarcinoma. In an interim analysis of safety data from a PMS study in Japanese patients with rheumatoid arthritis, a total of 25 patients reported all-cause malignancies (IR 1.25; 4874 PY of exposure), of which five patients had lymphoma/lymphoproliferative disorder and three patients had breast cancer [23]. Moreover, in an analysis of PMS data in patients with rheumatoid arthritis (439,370 PY of exposure) and psoriatic arthritis (20,706 PY of exposure), there were reports of lung neoplasm malignant, colon cancer, basal cell carcinoma, and squamous cell carcinoma [24], and in another PMS study in patients with rheumatoid arthritis (3247.6 PY of exposure), the most common malignancies reported were lung cancer, lymphoma and other lymphoproliferative disorders, and colon/rectal cancer [18].

Overall, two cardiovascular events were reported, specifically one subarachnoid hemorrhage and one subdural hematoma. The patient with subarachnoid hemorrhage was aged 53 years with no cardiovascular comorbidities, and the patient with subdural hematoma was aged 46 years and had a medical history of diverticula. Both cardiovascular events were not adjudicated and the physician in-charge ruled that there was no causal relationship. There were no cardiovascular events reported in those over 65 years old; one patient had a smoking history. One patient died following an intestinal abscess; this was a 79-year-old female who had a medical history of a digestive tract disorder and tuberculosis. In addition, this patient had comorbidities of diverticula, hypertension, lung damage, osteoporosis, and sleeplessness, and was receiving concomitant medication of iscotin and mesalazine.

Five venous thromboembolism AEs and three ADRs were reported, which included thrombophlebitis, venous thrombosis limb, and deep vein thrombosis. These were classified as venous thromboembolism due to the usage of the Preferred Terms in the Standardized MedDRA Query. The IR for venous thromboembolism in this analysis was 0.31 (95% CI 0.10, 0.73; 1592.23 PY of exposure), which was consistent with a previous real-world analysis of Japanese patients with inflammatory bowel disease in the Japan Medical Data Center claims database that reported a venous thromboembolism IR of 0.40 (95% CI 0.35, 0.47) in patients with UC [25], and numerically higher than the IR of deep vein thrombosis (IR 0.04 [95% CI 0.00, 0.23; 2403.6 PY of exposure]) and pulmonary embolism (IR 0.16 [95% CI 0.04, 0.41; 2403.6 PY of exposure]) reported in the tofacitinib UC clinical program [26].

In this PMS study, approximately half of the patients (52.4%) had discontinued tofacitinib treatment by week 60. The most common reason for treatment discontinuation was inadequate clinical response, which has also been found to be one of the most common reasons for discontinuation in multiple other cohorts of tofacitinib-treated patients with UC and rheumatoid arthritis (59% and 53.6%, respectively) [27, 28].

Effectiveness was observed throughout the PMS study, with most evaluable patients being in partial Mayo score remission at week 60. Previous studies have reported contradictory results on the potential effect of age on treatment effectiveness. In a longitudinal study of Spanish patients with UC who were in clinical remission (partial Mayo score ≤ 2), a univariate analysis found that age was not significantly associated with an increased risk of disease relapse [29]. In contrast, in a post hoc analysis of a Phase 3, randomized controlled trial assessing maintenance effectiveness in patients treated with golimumab, a multivariate regression analysis showed that it was more difficult for patients to achieve mucosal healing with increasing age, and this measure of effectiveness was not achieved by any patients over 60 years of age [30].

The strengths of this PMS study included the real-world setting and the large number of patients that were analyzed, which was most likely the largest cohort used to-date to evaluate the safety of tofacitinib. In addition, using an all-case surveillance approach further strengthened this study. Moreover, even though the study was conducted during the COVID-19 pandemic, the rate of serious infection AEs remained low. The PMS study also had some limitations. It was an observational study and healthcare professionals could adjust doses at their discretion; therefore, the analysis was limited in identifying the correlation between ADRs/AEs and tofacitinib dose. In addition, safety events were not centrally adjudicated. Moreover, the number of patients with evaluable total Mayo scores was small, which is partly due to the low number of patients with an endoscopic subscore that could be calculated.

In conclusion, the final results from the PMS analysis were generally consistent with the previously published results from the tofacitinib OCTAVE studies and the previous interim analyses of this PMS study. The overall safety profile during the treatment period was generally comparable with tofacitinib safety data previously reported in PMS reports from Japan and worldwide, and in the tofacitinib UC clinical program. However, rates of HZ were higher in this analysis than in the tofacitinib UC clinical program. The rates of AEs for tofacitinib reported in this analysis were lower when compared with data reported for other Janus kinase inhibitors and biologics used for the treatment of UC. Effectiveness of tofacitinib in Japanese patients with UC in a clinical practice setting was similar to that previously reported in the tofacitinib UC clinical program, with most patients with evaluable partial Mayo scores in remission during the 60-week observation period. Overall, no new safety concerns were identified and these results suggest that tofacitinib is effective in patients with UC in Japan.

## Supplementary Information

Below is the link to the electronic supplementary material.

Supplementary file1 (PDF 372 KB)

## Data Availability

Upon request, and subject to review, Pfizer will provide the data that support the findings of this study. Subject to certain criteria, conditions, and exceptions, Pfizer may also provide access to the related individual de-identified participant data. See https://www.pfizer.com/science/clinical-trials/trial-data-and-results for more information.

## Abbreviations

ADR: Adverse drug reaction
AE: Adverse event
CI: Confidence interval
HZ: Herpes zoster
IR: Incidence rate
MedDRA: Medical Dictionary for Regulatory Activities
NRI-LOCF: Non-responder imputation-last observation carried forward
PMS: Post-marketing surveillance
PY: Patient-years
SD: Standard deviation
UC: Ulcerative colitis
