# Supplementary material for: Post-marketing surveillance of tofacitinib in patients with ulcerative colitis in Japan: a final report of safety and effectiveness data
Source: J Gastroenterol. 2025 Apr 21;60(8):979–89. doi: 10.1007/s00535-025-02249-5 (PMC12289845; doi:10.1007/s00535-025-02249-5)
Supplement: Supplementary file 1 — Supplementary file1 (PDF 372 KB) [file 535_2025_2249_MOESM1_ESM.pdf]

Supplementary Material

Article title: Post-marketing surveillance of tofacitinib in patients with ulcerative colitis in Japan: a final report of safety and effectiveness data

Journal name: Journal of Gastroenterology

Author names: Katsuyoshi Matsuoka[1,2], Satoshi Motoya[2,3], Takayuki Yamamoto[2,4], Minoru Matsuura[2,5], Toshimitsu Fujii[2,6], Shinichiro Shinzaki[2,7], Yohei Mikami[2,8], Shoko Arai[9], Junichi Oshima[10], Yutaka Endo[10], Hirotoshi Yuasa[10], Masato Hoshi[9], Keiko Sato[10], Tadakazu Hisamatsu[2,5]

Affiliations:

[1]*Division of Gastroenterology and Hepatology, Department of Internal Medicine, Toho University Sakura Medical Center, Chiba, Japan*

[2]*Clinical Epidemiology Committee of the Japanese Society for Inflammatory Bowel Disease*

[3]*Inflammatory Bowel Disease Center, Sapporo-Kosei General Hospital, Hokkaido, Japan*

[4]*Department of Surgery and IBD Center, Yokkaichi Hazu Medical Center, Yokkaichi, Mie, Japan*

[5]*Department of Gastroenterology and Hepatology, Kyorin University, Mitaka, Tokyo, Japan*

[6]*Department of Gastroenterology and Hepatology, Tokyo Medical and Dental University, Tokyo, Japan*

[7]*Department of Gastroenterology, Faculty of Medicine, Hyogo Medical University, Nishinomiya, Hyogo, Japan*

[8]*Division of Gastroenterology and Hepatology, Department of Internal Medicine, Keio University School of Medicine, Tokyo, Japan*

[9]*Pfizer Japan Inc, Tokyo, Japan*

[10]*Pfizer R&D Japan, Tokyo, Japan*

Corresponding author: Shoko Arai, Pfizer Japan Inc, 3-22-7 Yoyogi, Shibuya-ku, Tokyo, Japan. Shoko.Arai@pfizer.com

Supplementary Table 1 Proportions and IRs of ADRs in the PMS study[a]

Clinically important/potential risks[b]	Adverse drug reaction[b]		
	n (%)	Exposure, PY	IR (95% CI)
HZ (non-serious and serious)	89 (4.5)[c]	1554.05	5.73 (4.60, 7.05)
Dyslipidemia	77 (3.9)[d]	1535.81	5.01 (3.96, 6.27)
Neutropenia/lymphopenia/hemoglobin decreased	38 (1.9)[e]	1571.78	2.42 (1.71, 3.32)
Rhabdomyolysis/myopathy	19 (1.0)[f]	1580.21	1.20 (0.72, 1.88)
Serious infection	18 (0.9)[g]	1590.60	1.13 (0.67, 1.79)
Liver dysfunction	12 (0.6)[h]	1586.67	0.76 (0.39, 1.32)
Malignancy	7 (0.4)[i]	2738.99	0.26 (0.10, 0.53)
Venous thromboembolism	3 (0.2)[j]	1592.79	0.19 (0.04, 0.55)
Interstitial lung disease	2 (0.1)[k]	1592.83	0.13 (0.02, 0.45)
Gastrointestinal perforation	1 (0.1)[l]	1593.82	0.06 (0.00, 0.35)
Reactivation of hepatitis B virus	0[m]	19.75	0.00[n]
Cardiovascular events	0[o]	1594.12	0.00[n]

All events were non-adjudicated and judged by the investigators. The observation period was defined as the tofacitinib treatment period, which was the period from the date of tofacitinib initiation up to 60 weeks after the start date or up to June 2021. Patients who were treated with tofacitinib for more than 60 weeks after the date of treatment commencement were observed until June 2021 (end of the observation period; data as of September 30, 2022). All ADRs within 60 weeks or until the end of the observation period (whichever came sooner) were recorded. Malignancies were recorded until the end of the observation period.

[a]Data reported during the treatment period, as of September 30, 2022; 1982 patients were included in the safety analysis (investigator-reported Preferred Term MedDRA v.24.1).

[b]An adverse reaction was defined as an adverse event for which a causal relationship with tofacitinib

could not be ruled out. An adverse event was defined as any unfavorable event (including a clinically significant abnormal laboratory change) occurring after administration of tofacitinib, whether related to tofacitinib or not.

[c]Four events (0.2%) were serious.

[d]Metabolism and nutrition disorders: hyperlipidemia (*n*=28); dyslipidemia (*n*=17); hypercholesterolemia (*n*=16); and hypertriglyceridemia (*n*=5). Congenital, familial and genetic disorders: type IIa hyperlipidemia (*n*=1). Investigations: lipids abnormal (*n*=4); blood cholesterol increased and low-density lipoprotein increased (both *n*=3); and blood triglycerides increased (*n*=2).

[e]Blood and lymphatic system disorders: anemia (*n*=17); and anemia macrocytic (*n*=1). Investigations: lymphocyte count decreased (*n*=12); neutrophil count decreased (*n*=8); and hemoglobin decreased and red blood count cell decreased (both *n*=1).

[f]Musculoskeletal and connective tissue disorders: rhabdomyolysis (*n*=1). Investigations: blood creatine phosphokinase increased (*n*=18).

[g]Infections and infestations: HZ (*n*=4); cytomegalovirus infection, pyelonephritis, pneumonia and atypical mycobacterial infection (all *n*=2); and cytomegalovirus enterocolitis, cytomegalovirus chorioretinitis, eczema herpeticum, infection, bone tuberculosis and sepsis (all *n*=1).

[h]Hepatobiliary disorders: liver disorder (*n*=9). Investigations: alanine aminotransferase increased (*n*=2); and aspartate aminotransferase increased and hepatic enzyme increased (both *n*=1).

[i]Neoplasm malignant and testis cancer (both *n*=2); and adenocarcinoma and pancreatic carcinoma (both *n*=1). Investigations: carcinoembryonic antigen increased (*n*=1).

[j]Venous thromboembolism events were identified using Preferred Terms in the Standardised MedDRA Query ‘Embolic and thrombotic events, venous’: thrombophlebitis (*n*=2) and venous thrombosis limb (*n*=1).

[k]Respiratory, thoracic and mediastinal disorders: eosinophilic pneumonia and interstitial lung disease (both *n*=1).

[l]Anal fistula (*n*=1).

[m]Only patients with a history of hepatitis B virus or complications of hepatitis B virus carrier were included (*n*=23); ‘Exposure, PY’ is based on these patients only.

[n]95% CI not calculated.

[o]One adverse event of subarachnoid hemorrhage and one adverse event of subdural hematoma were reported.

ADR, adverse drug reaction; CI, confidence interval; HZ, herpes zoster; IR, incidence rate (number of unique patients with events per 100 PY of exposure); MedDRA, Medical Dictionary for Regulatory Activities; n, number of patients with the specified event; *n*, number of cases; PMS, post-marketing surveillance; PY, patient-years.

Supplementary Table 2 Risk factor analysis results for association of each baseline variable with HZ ADRs in the PMS study[a]

**Factor**	**HR (95% CI)**
Sex	
Female vs male	1.16 (0.76, 1.76)
Age	
≥ 50 to < 65 years vs < 50 years	1.13 (0.69, 1.87)
**≥ 65 years vs < 50 years**	**1.98 (1.14, 3.44)**
Duration of ulcerative colitis	
≥ 2 to < 5 years vs < 2 years	0.89 (0.46, 1.74)
≥ 5 to < 10 years vs < 2 years	1.18 (0.63, 2.24)
≥ 10 to < 20 years vs < 2 years	1.29 (0.69, 2.42)
≥ 20 years vs < 2 years	0.80 (0.31, 2.05)
Smoking history	
Former smoker vs never smoked	1.34 (0.82, 2.18)
Current smoker vs never smoker	0.98 (0.39, 2.44)
History of infection	
**Yes vs no**	**4.87 (2.59, 9.16)**
Prior corticosteroid use[b]	
Yes vs no	0.95 (0.63, 1.44)
Prior biologic use	
**Yes vs no**	**1.69 (1.02, 2.80)**

Factors associated with an increased risk of HZ in the univariate analysis are shown in **bold text.**

[a]Data reported during the treatment period, as of September 30, 2022; 1982 patients were included in the safety analysis (up to 2706.4 PY of exposure).

[b]History of treatment for the episode immediately before the commencement of tofacitinib treatment.

CI, confidence interval; HR, hazard ratio; HZ, herpes zoster; PMS, post-marketing surveillance; PY, patient-years.

Supplementary Fig. 1 Patient disposition in the PMS study.

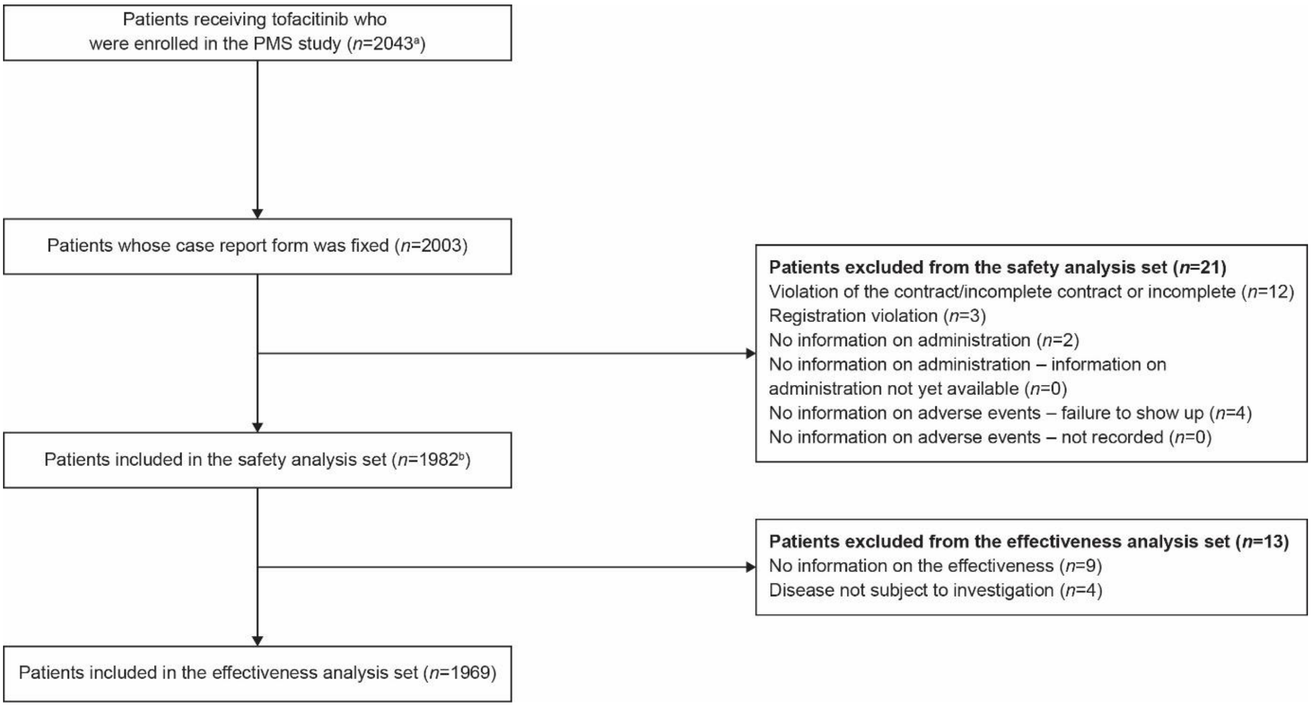

[a]Data were excluded for 13 patients from two hospitals from which consent was not obtained and which were therefore not permitted for publication.

[b]Four patients with pouchitis were included in the safety analysis set; tofacitinib is not indicated for pouchitis treatment.

PMS, post-marketing surveillance.

Supplementary Fig. 2 **a** Proportion of patients in remission by partial Mayo score and total Mayo score, and **b** mean changes from baseline in partial Mayo score and total Mayo score, up to Week 60 in the PMS study[a] (as observed).

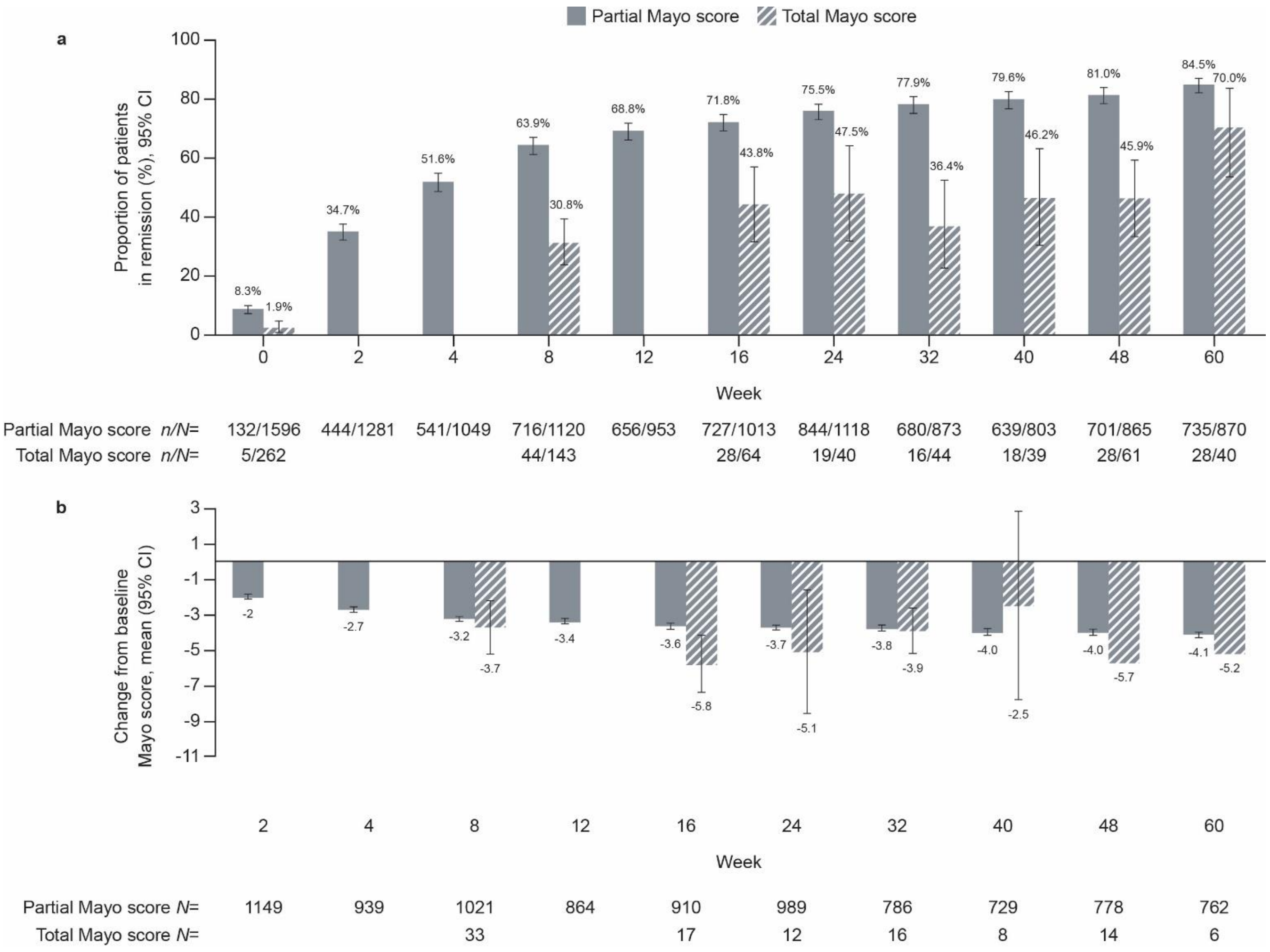

The observation period was defined as the tofacitinib treatment period, which was the period from the date of tofacitinib initiation up to 60 weeks after the start date or up to June 2021. Patients who were treated with tofacitinib for more than 60 weeks after the date of treatment commencement were continuously observed until June 2021 (end of the observation period). Remission by partial Mayo score was defined as Mayo score of ≤ 2 points, stool frequency subscore of ≤ 1 point, rectal bleeding subscore of ≤ 1 point, and PGA subscore of ≤ 1 point. Remission by total Mayo score was defined as total Mayo score of ≤ 2 points, every subscore of ≤ 1 point, and rectal bleeding subscore of 0 points. The remission rate was defined as the proportion of patients in remission among those with evaluable partial Mayo score/total Mayo scores in the effectiveness analysis set.

[a]Data reported during the treatment period; 1969 patients were included in the analysis of tofacitinib effectiveness.

CI, confidence interval; *n*, number of patients in remission by partial Mayo score/total Mayo score; *N*, number of the patients whose partial Mayo score/total Mayo score could be evaluated; PGA, Physician Global Assessment; PMS, post-marketing surveillance.
